# Extraplatelet Molecule Enrichment Enhances the Ability of Platelet-Rich Plasma Releasate to Buffer IL-1β-Induced Inflammation and Oxidative Stress in a Primary Human Chondrocyte–Synoviocyte Co-Culture In Vitro System of Osteoarthritis

**DOI:** 10.3390/ijms27208950

**Published:** 2026-10-09

**Authors:** Daniel Marijuán-Pinel, Jon Mercader-Ruiz, Xabier Sansinanea, Pello Sánchez, Renato Andrade, João Espregueira-Mendes, Leonor López de Dicastillo, Diego Delgado, Mikel Sánchez

**Affiliations:** 1 Advanced Biological Therapy Unit, MiKS Hospital, 01010 Vitoria-Gasteiz, Spain; daniel.marijuan@hospitalmiks.com (D.M.-P.); jon.mercader@hospitalmiks.com (J.M.-R.); pello.sanchez@hospitalmiks.com (P.S.); diego.delgado@hospitalmiks.com (D.D.); 2 Arthroscopic Surgery Unit, MiKS Hospital, 01010 Vitoria-Gasteiz, Spain; xabi.sansinanea@hospitalmiks.com (X.S.); leonor.lopez@hospitalmiks.com (L.L.d.D.); 3 Clínica Espregueira—FIFA Medical Centre of Excellence, 4350-415 Porto, Portugal; 4 Dom Henrique Research Centre, 4350-415 Porto, Portugal; 5 Porto Biomechanics Laboratory (LABIOMEP), Faculty of Sports, University of Porto, 4200-450 Porto, Portugal; 6 School of Medicine, University of Minho, 4710-057 Braga, Portugal; 7 ICVS/3B′s-PT Government Associate Laboratory, 4710-057 Braga, Portugal; 8 3B′s Research Group—Biomaterials, Biodegradables and Biomimetics, University of Minho, Headquarters of the European Institute of Excellence on Tissue Engineering and Regenerative Medicine, 4806-909 Barco, Portugal

**Keywords:** platelet-rich plasma, osteoarthritis, primary chondrocyte–synoviocyte co-culture, inflammation, oxidative stress, extracellular matrix remodeling

## Abstract

To evaluate the anti-inflammatory and antioxidant effects of standard platelet-rich plasma (sPRP) and balanced protein-concentrate plasma (BPCP), which is a next-generation formulation enriched in platelet-derived and extraplatelet bioactive molecules, in an indirect co-culture model of primary human chondrocytes (hCh) and synoviocytes (hSv) under IL-1β-induced inflammatory conditions. hCh and hSv were co-cultured in a transwell system (indirect co-culture) and stimulated with IL-1β (10 ng/mL) for 48 h in the presence of 10% sPRP, 10% BPCP, or serum-free medium (SF). Gene expression in hCh was analyzed by RT-qPCR for inflammatory mediators, NF-κB pathway genes, matrix-degrading enzymes and apoptosis markers. Cytokine and MMP secretion in supernatants were quantified by Luminex assay, and intracellular ROS levels were measured in hSv and hCh direct co-culture by DCFDA fluorescence after 4 h of incubation with PRP previously exposed for 24 h to IL-1β. Both sPRP and BPCP significantly reduced MCP-1 (*p* = 0.02) and BCL-2 expression (*p* < 0.0001) versus SF. Both formulations also decreased secretion of IL-1β, TNF-α, IL-6, IL-8, MCP-1, MMP-1, MMP-3, and MMP-13 compared to SF. BPCP showed greater reductions in IL-6 and MCP-1 compared with sPRP. MMP-7 and MMP-9 secretion increased under both PRP conditions, consistent with tissue remodeling activity. Both formulations significantly reduced intracellular ROS levels measured by DCFDA fluorescence, with a stronger effect observed for BPCP. sPRP and BPCP exerted consistent anti-inflammatory and antioxidant effects in a multicellular simulated osteoarthritic in vitro model. BPCP demonstrated enhanced protein-level regulation in certain markers, suggesting that enrichment in extraplatelet molecules may improve PRP efficacy.

## 1. Introduction

Osteoarthritis (OA) is a leading cause of chronic pain and functional disability worldwide, with prevalence continuing to rise in association with aging populations, obesity, joint injury, and genetic susceptibility [1,2]. Its pathogenesis is driven by complex, multidirectional interactions between mechanical, cellular, and biochemical factors, in which chondrocytes and synoviocytes play a central and interrelated role [3,4]. Chondrocytes are responsible for maintaining the homeostasis of the extracellular matrix (ECM), which, under the inflammatory conditions of OA, adopts a catabolic phenotype. Simultaneously, synoviocytes amplify the inflammatory cascade by secreting pro-inflammatory cytokines and matrix-degrading enzymes, creating a pathological microenvironment that accelerates joint destruction [5,6].

Current therapeutic options remain predominantly symptomatic and fail to halt disease progression. This has led to interest in biologic therapies capable of modulating the joint microenvironment [7]. Among these, platelet-rich plasma (PRP) has emerged as a promising autologous and biological therapy for OA [8,9]. This blood-derived product, enriched in platelets, releases a wide range of bioactive molecules such as platelet-derived growth factor (PDGF), transforming growth factor-β1 (TGF-β1), and vascular endothelial growth factor (VEGF) [10,11,12,13]. Preclinical studies have demonstrated that PRP promotes chondrocyte anabolism while suppressing catabolic signaling through downregulation of IL-1β- and TNF-α-mediated pathways, MMP/ADAMTS expression, and NF-κB activation, with additional antioxidant and anti-senescent effects on joint cells [14,15,16,17,18]. These findings are supported by clinical evidence showing meaningful symptomatic and functional improvement following intra-articular PRP injection in early-to-moderate OA, positioning PRP as a potential disease-modifying biological therapy [19,20,21].

However, a critical challenge in the field remains the lack of consensus regarding optimal PRP composition. Standard preparations (sPRP) concentrate platelet-derived molecules, yet growing evidence [22] suggests that extraplatelet circulating factors may play an equally important modulatory role [23]. Indeed, it has been demonstrated that extraplatelet molecules, such as insulin-like growth factor (IGF-1) and hepatocyte growth factor (HGF), influence tissue repair and inflammation [23,24,25,26,27]. Next-generation preparations, such as the balanced protein-concentrate plasma (BPCP), achieve simultaneous enrichment of both platelet and extraplatelet bioactive molecules, doubling HGF and IGF-1 levels, showing an improvement in cell viability and anti-inflammatory effects [28,29].

A parallel challenge lies in the in vitro models used to investigate PRP efficacy. Traditional monoculture systems fail to reproduce the intercellular interactions that are fundamental to the OA pathophysiology, in which paracrine communication between chondrocytes and synoviocytes sustains the inflammatory and catabolic environment [30,31,32,33]. Therefore, co-culture systems enable the study of cell–cell communication under controlled conditions while preserving greater physiological relevance [34,35,36]. The integration of PRP treatment with co-culture models offers a promising approach to better understand its mechanisms and to optimize its use as a biological therapy.

In the present study, we employed a transwell-based in vitro co-culture model of primary human chondrocytes (hCh) and synoviocytes (hSv) stimulated with IL-1β to simulate the OA inflammatory microenvironment, and compared the biological effects of sPRP and BPCP. This work was designed as an initial screening study to validate the co-culture model as a platform for investigating PRP in vitro bioactivity in OA, and to determine whether the enrichment of extraplatelet molecules in BPCP confers major therapeutic efficacy, thereby informing the rational optimization of PRP-based biological therapies.

## 2. Results

### 2.1. Platelet Level Measurement

Platelet levels were measured in PC, sPRP, and BPCP to compare their biochemical composition. Platelet analysis showed that the platelet content in sPRP (452 × 10^3^ platelets/µL ± 63.38) and BPCP (468 × 10^3^ platelets/µL ± 60.65) was twice that of blood basal levels (235.5 × 10^3^ platelets/µL ± 41.78), and these differences were statistically significant (** *p* = 0.001 and *** *p* = 0.0006, respectively). Moreover, there was no significant difference in platelet concentration between sPRP and BPCP (*p* = 0.915). Meanwhile, residual leukocytes and erythrocytes were below the detection limit. According to the UCS (Universal Coding System) for PRP studies described by Kon et al. [37], the products used in this study were 24-00-11 (Table 1).

### 2.2. PRP Modulates Gene Expression in Human Primary Chondrocytes Under Inflammatory Stimulation

**Figure 1:**
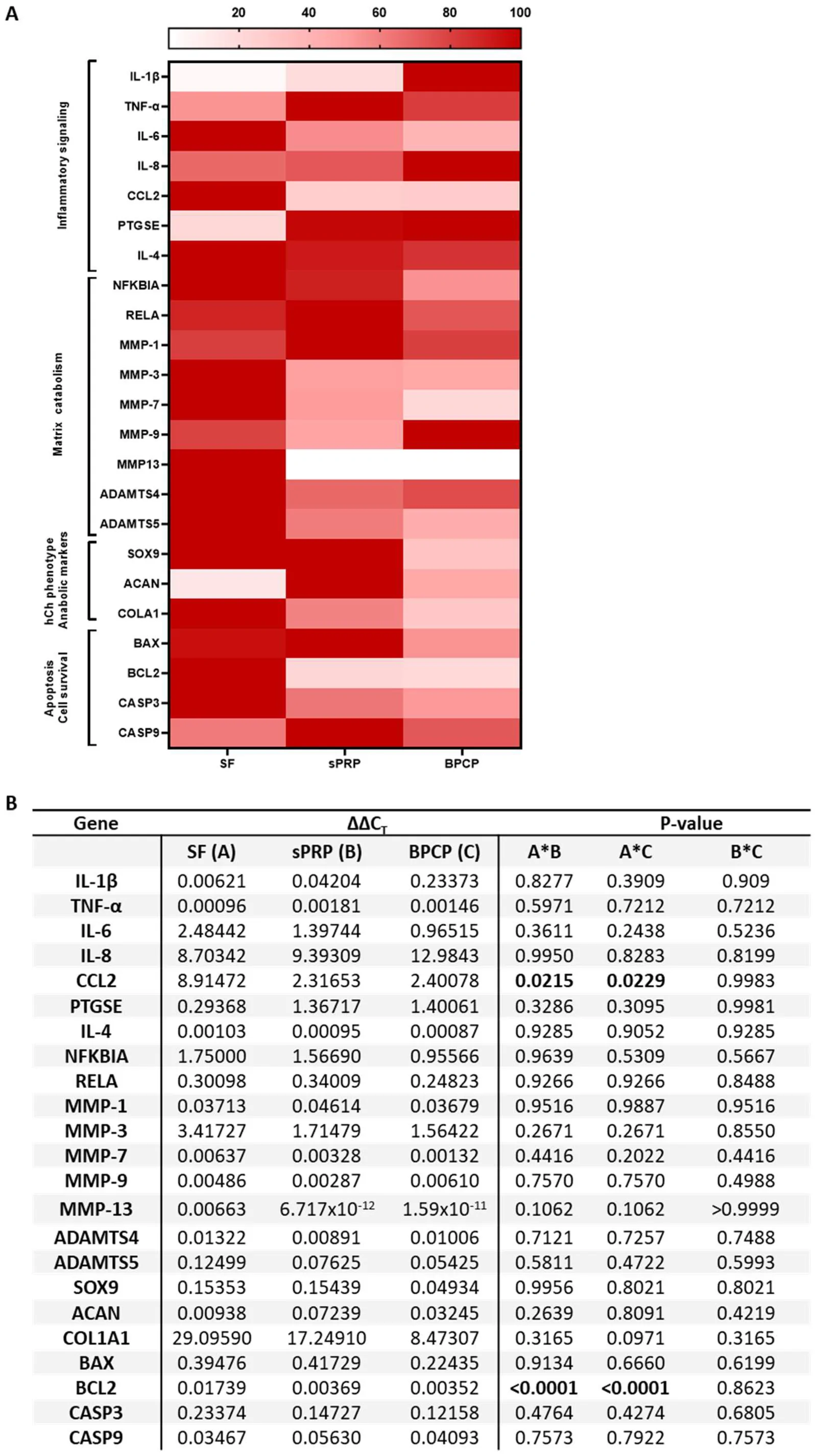
Gene expression analysis in hCh following simultaneous stimulation with IL-1β and treatment with PRP formulations. Relative gene expression levels were determined by RT-qPCR in hCh after 48 h of transwell co-culture with hSv in the presence of IL-1β (10 ng/mL) and SF, sPRP, or BPCP releasates at 10% (*v*/*v*). Gene expression was normalized to housekeeping genes and calculated using the ΔΔCt method. (**A**) Heat map of analyzed genes, including inflammatory mediators, signaling pathway regulators, matrix-degrading enzymes, extracellular matrix-related genes and apoptosis-related genes. (**B**) Table with ΔΔCt values and statistics for each gene expression. Error bars = standard deviation (biological n = 4), with 2 technical replicates. Statistically significant differences were calculated using one-way ANOVA. Statistics marked in bold represent statistical significance between groups (A, B or C).

To evaluate the biological effects of sPRP and BPCP formulations on OA-related molecular pathways, gene expression was analyzed in hCh following a transwell co-culture with hSv under IL-1β stimulation (Figure 1A,B). IL-1β stimulation induced a broad upregulation of inflammatory gene expression in hCh, recreating an inflammatory environment similar to that seen in OA. Treatment with both sPRP and BPCP was associated with a consistent downward trend in the expression of pro-inflammatory mediators, although most changes did not reach statistical significance, likely reflecting the limited statistical power inherent to the pooled experimental design. The most notable exception was MCP-1, which showed a statistically significant reduction in both PRP-treated groups relative to the SF condition (*p* = 0.02 for both). A similar trend toward reduced expression was observed for IL-6, while IL-1β, IL-8, TNF-α, and PTGS2 did not follow this pattern and showed marginally elevated values under PRP treatment. Similarly, genes associated with NF-κB signaling, such as NFKBIA and RELA, showed a trend toward reduced expression following treatment, particularly with BPCP.

ECM-degrading enzymes showed treatment-dependent modulation with variable patterns across MMP subtypes. MMP-3 tended toward lower expression in both PRP-treated groups relative to SF cultures, while MMP-1 remained highly expressed across all conditions. MMP-9 showed a moderate reduction with sPRP but not with BPCP, whereas MMP-13 expression was markedly reduced or undetectable in both PRP-treated groups. ADAMTS4 and ADAMTS5 showed a consistent, although non-significant, trend toward reduced expression in PRP-treated conditions relative to SF.

Regarding ECM-related genes, expression of SOX9, ACAN, and COL1A1 showed distinct trends. SOX9 expression was high in both SF and sPRP conditions but decreased in the BPCP group. In contrast, ACAN expression increased markedly in the sPRP condition compared with SF cultures and showed intermediate levels in BPCP. COL1A1 expression was highest in the SF condition and decreased in both PRP treatments, particularly in the BPCP group, although these differences did not reach statistical significance.

Apoptosis-related genes showed treatment-dependent modulation. BCL-2 expression was significantly reduced in both PRP-treated groups compared with the SF condition (*p* < 0.0001). BAX showed a trend toward increased expression with sPRP but lower levels with BPCP, while CASP3 tended toward reduced expression in both PRP conditions relative to SF. CASP9 showed marginally elevated expression in the sPRP group, though without statistical significance.

### 2.3. PRP Reduces the Secretion of OA-Related Proteins Under Inflammatory Conditions

**Figure 2:**
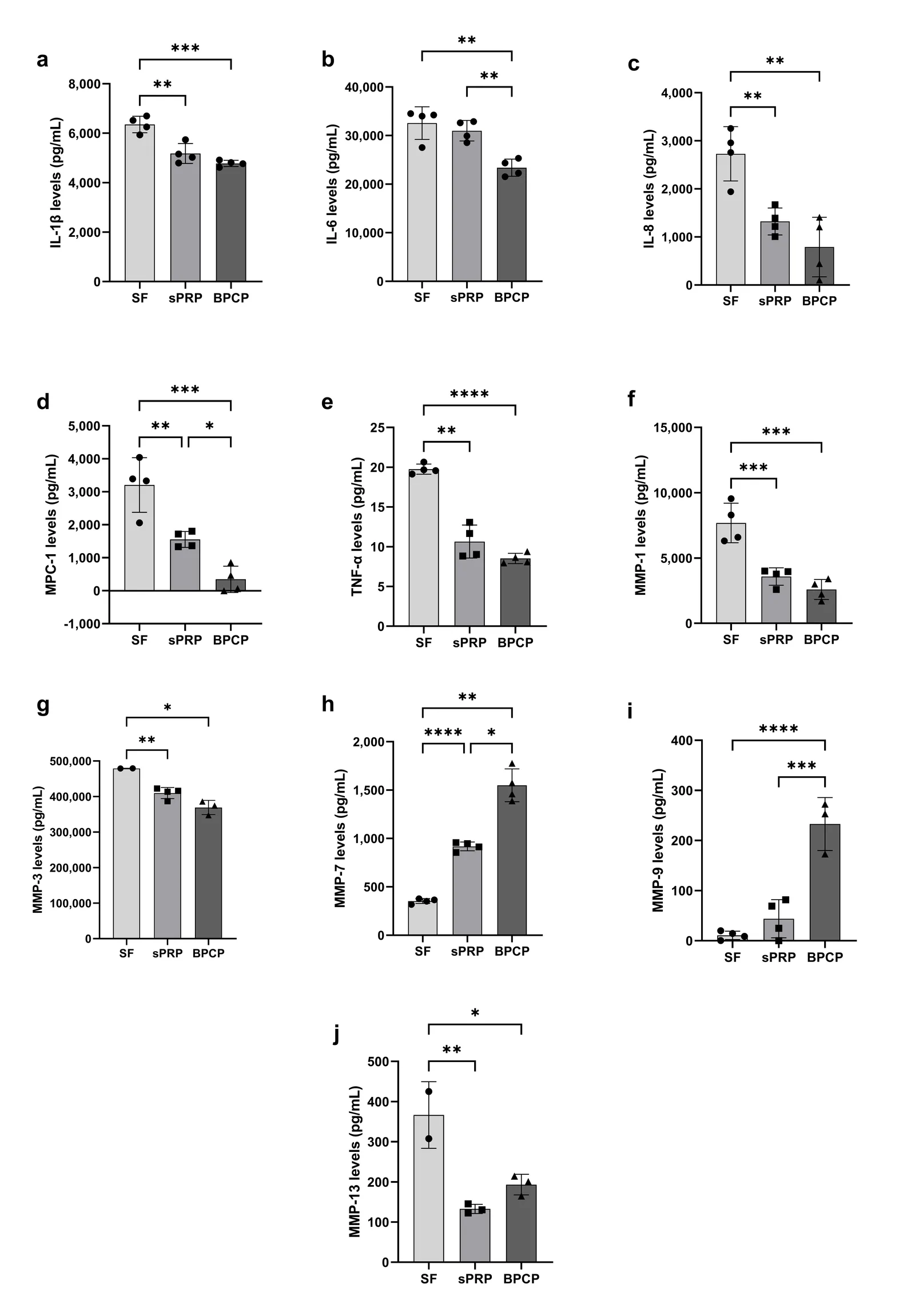
Modulation of secretion of cytokines and proteins in hCh-hSv transwell co-culture in the presence of IL-1β. Concentration of (**a**) IL-1β, (**b**) IL-6, (**c**) IL8, (**d**) MCP-1, (**e**) TNF-α, (**f**) MMP-1, (**g**) MMP-3, (**h**) MMP-7, (**i**) MMP-9, and (**j**) MMP-13 under inflammatory conditions in cell supernatant treated with SF, sPRP, or BPCP releasates at 10% (*v*/*v*) after 48 h. Results are expressed as the difference in protein concentration between IL-1β-exposed and unexposed cells, except for MMP-7 and MMP-13, for which only values under inflammatory conditions are shown. Error bars = standard deviation (biological n = 4), with four technical replicates. Statistically significant differences were calculated using one-way ANOVA and Welch ANOVA test (* *p* < 0.05; ** *p* < 0.01; *** *p* < 0.001; **** *p* < 0.0001).

Protein secretion analysis was performed on transwell co-culture supernatants to assess the effects of both PRP formulations on inflammatory signaling at the protein level. Because sPRP and BPCP contain different concentrations of soluble bioactive molecules, baseline protein concentrations were quantified in both IL-1β-exposed and unexposed conditions (Supplementary Materials, Tables S2 and S3). To account for these treatment-dependent baseline differences, the results presented in Figure 2 are expressed as the change in protein concentration between IL-1β-exposed and corresponding unexposed conditions within each treatment group. The exceptions were MMP-7 and MMP-13, as their levels were below the detection limit under unstimulated conditions. This analysis was intended to quantify the protein-level response associated with IL-1β exposure rather than to distinguish cellular proteins from proteins intrinsically present in the PRP preparations.

Under this approach, treatment with both sPRP and BPCP was associated with a significant attenuation of several pro-inflammatory mediators relative to SF. Specifically, IL-1β, IL-8, MCP-1, and TNF-α were reduced in both PRP-treated groups, with BPCP additionally showing a statistically significant reduction in IL-6 and MCP-1 secretion compared with sPRP (Figure 2b,d). IL-4 did not differ significantly between groups, and IL-10 was below the detection limit across all conditions.

Similarly, several MMPs associated with cartilage degradation, such as MMP-1, MMP-3, and MMP-13 (Figure 2f,g,j), showed statistically significant reduced secretion levels after treatment with both PRPs relative to SF. Regarding other MMPs, MMP-7 and MMP-9 (Figure 2h,i) showed a statistically significant increase in their concentration for both PRP types.

Overall, IL-1β, IL-8, TNF-α, MMP-1, and MMP-3 showed that BPCP has a trend toward lower levels compared to sPRP.

### 2.4. PRP Decreases Intracellular ROS in hCh and hSv Cell Co-Culture

**Figure 3:**
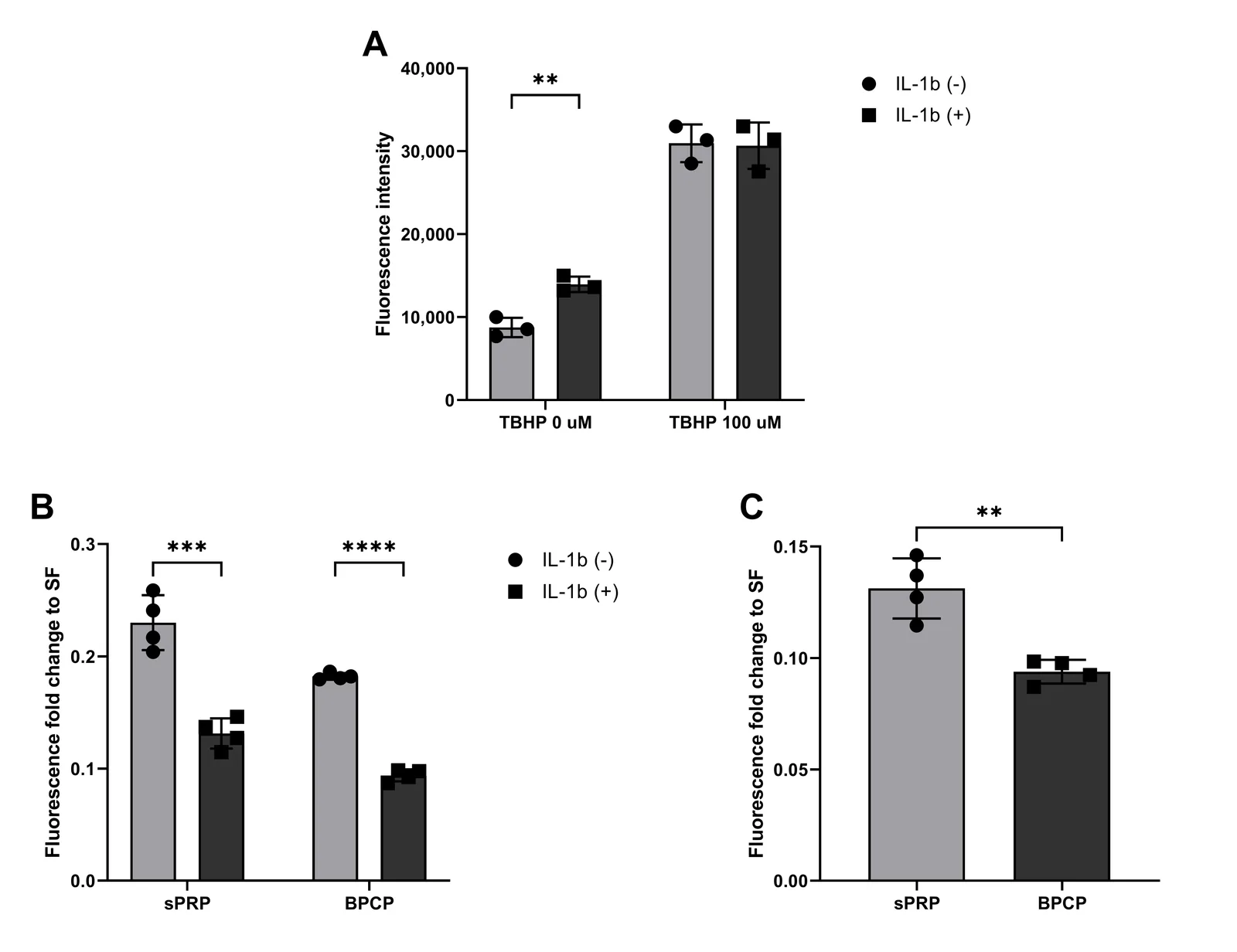
Intracellular reactive oxygen species (ROS) measured using the DCFDA assay in hCh and hSv direct in vitro co-culture. Cells were exposed to 10 ng/mL IL-1β in complete CM for 24 h. After that, cells were treated with sPRP or BPCP releasates at 10% (*v*/*v*) in Buffer 1× for 4 h. (**A**) ROS measurement in cells exposed or not to IL-1β after treatment with Buffer 1× for 4 h. Cells treated with 100 uM of TBHP were used as a positive control. (**B**) ROS fluorescence fold-change in sPRP and BPCP, both in cells exposed and not exposed to IL-1β. (**C**) ROS fluorescence fold-change to SF of sPRP and BPCP after being exposed to IL-1β. Data was obtained from four independent PRP pools (biological n = 4), with three technical replicates. All samples were carried out in triplicate. Data are shown as mean fold change to the SF control in Buffer 1× ± SD. Statistically significant differences were calculated using Student’s *t* test: ** *p* < 0.01, *** *p* < 0.001, and **** *p* ≤ 0.0001.

To assess the antioxidant potential of both PRP formulations, intracellular ROS levels were measured using the DCFDA assay in a direct hCh-hSv in vitro co-culture model. As shown in Figure 3A, exposure to IL-1β for 24 h significantly increased intracellular ROS production compared with unexposed cells, confirming the establishment of an oxidative stress environment; TBHP was included as a positive control to validate the assay sensitivity. Under these inflammatory conditions, both sPRP and BPCP showed lower fluorescence signals relative to the SF condition in IL-1β-exposed cells (Figure 3B). Treatment with either sPRP or BPCP for 4 h showed significantly reduced intracellular ROS levels. This reduction was more pronounced in BPCP-treated cells (Figure 3).

## 3. Discussion

The present study demonstrates that both sPRP and BPCP exert consistent anti-inflammatory and antioxidant effects in an in vitro co-culture model of hCh and hSv cells under IL-1β stimulation. By integrating gene expression, protein secretion, and intracellular ROS analyses, as a first screening step, we show that both formulations modulate the simulated OA environment in vitro, with BPCP exhibiting a trend toward a more pronounced regulatory profile.

At the transcriptional level, PRP treatment was associated with a consistent downward trend in pro-inflammatory gene expression in IL-1β-stimulated hCh. Notably, a statistically significant reduction in MCP-1 was observed in both PRP-treated groups, which is particularly relevant given its central role in monocyte recruitment and synovial inflammation amplification in OA [38]. Although most changes did not reach statistical significance, the marginal upregulation of IL-1β, IL-8, TNF-α, and PTGS2 may reflect a biphasic behavior and transient early-phase activation preceding a subsequent resolution phase, as previously described in PRP-treated biological systems [39,40,41].

The trend toward reduced expression of NF-κB-related genes (NFKBIA and RELA) further supports the hypothesis that both PRP formulations interfere with this central inflammatory signaling pathway, with a slight decrease observed for BPCP compared to sPRP. This is consistent with previous reports describing NF-κB inhibition as a key mechanism underlying the anti-inflammatory action of PRP, partly mediated by HGF, an extraplatelet factor particularly enriched in BPCP [29,41,42,43]. Given that NF-κB governs the transcription of pro-inflammatory cytokines and catabolic enzymes [44,45], moderate attenuation at this regulatory node may translate into amplified downstream effects at the protein level, which could help explain the more pronounced differences observed in the secretion data.

A particularly relevant finding emerged when comparing cytokine secretion profiles in the presence and absence of inflammatory stimulation. In a non-inflammatory setting, both sPRP and BPCP were associated with elevated baseline cytokine levels relative to SF, consistent with the intrinsic bioactive content of platelet-derived formulations and in line with previous reports describing an initial pro-inflammatory activation phase induced by PRP in the absence of pathological stimuli [29,46,47]. Nevertheless, under inflammatory conditions, statistically significant mitigation in the secretion of key pro-inflammatory mediators, including IL-1β, TNF-α, IL-6, IL-8, and MCP-1, in both PRP-treated groups relative to SF was observed. Moreover, BPCP additionally showed superior attenuation of IL-6 and MCP-1 compared with sPRP. This implies that, upon IL-1β addition, a markedly different pattern was observed: while SF-treated cells exhibited a pronounced amplification of cytokine release, PRP-treated cells showed only a modest increase, resulting in substantially attenuated net inflammatory output. This observation suggests that PRP does not act primarily by suppressing cytokine production per se, but rather by conditioning the cellular response to inflammatory challenge, effectively buffering the hyperinflammatory amplification that characterizes the OA synovial environment. The more pronounced differences at the protein versus transcriptional level suggest that PRP, under inflammatory conditions, might exert post-transcriptional regulatory activity, potentially modulating mRNA stability, translation efficiency, or protein secretion pathways, which is consistent with the known capacity of the bioactive milieu of PRP to influence cellular responses beyond direct transcriptional regulation [40].

The modulation of ECM-degrading enzymes revealed a biologically coherent pattern when gene expression and protein secretion data are considered together. At the protein level, both PRP formulations significantly reduced the secretion of MMP-1, MMP-3, and MMP-13, enzymes centrally involved in the degradation of collagen type I, ACAN, and collagen type II, respectively [48,49,50], with MMP-13 suppression in particular underscoring the chondroprotective potential of both treatments [15,41]. In contrast, MMP-7 and MMP-9 secretion was significantly increased, representing changes in matrix-remodeling markers that warrant further functional assessment of matrix turnover before attributing a definitive net effect [51]. Collectively, these findings highlight the MMP subtype-specific nature of PRP’s modulatory effects, with a net balance that favors cartilage protection over catabolism.

The reduced fluorescence signal may indicate a reduction in intracellular ROS levels observed following 24 h of incubation with IL-1β and 4 h treatment with both PRP formulations. Oxidative stress plays a well-established pathogenic role in OA, driving chondrocyte apoptosis, ECM degradation, and NF-κB activation in a self-amplifying loop that perpetuates joint destruction [52,53,54]. By attenuating ROS production under IL-1β-induced inflammatory conditions, with BPCP showing superior fluorescence reduction capacity, both formulations may interrupt this oxidative–inflammatory feedback at a fundamental level. This antioxidant effect carries particular mechanistic significance for two reasons. First, since oxidative stress regulates both transcriptional and post-transcriptional processes [55], its attenuation provides a plausible integrative explanation for the discrepancies observed between gene expression and protein secretion data throughout this study. PRP-mediated ROS reduction may globally dampen inflammatory signaling through mechanisms that are not fully captured at the mRNA level. Second, the reduction in ROS is consistent with the modulation of apoptosis-related genes observed in hCh, including the significant regulation of BCL-2 and the trend toward reduced CASP3 expression, suggesting that both formulations influence cellular pathways related to survival under the chronic oxidative stress conditions characteristic of the OA joint [18,56]. In fact, this reduction in BCL-2 could indicate a lack of activation of apoptotic pathways, but additional testing would be needed to confirm this. The more pronounced antioxidant activity of BPCP further supports the hypothesis that extraplatelet molecules could contribute meaningfully to the overall therapeutic profile of this formulation.

When comparing both formulations directly, BPCP showed a trend to a more pronounced regulatory profile relative to sPRP, most notably at the protein level, where statistically significant reductions in IL-6 and MCP-1 secretion were observed specifically in BPCP-treated cells. This enhanced effect is consistent with the previously characterized enrichment of extraplatelet circulating molecules in the BPCP formulation [28,29], which are hypothesized to synergize with platelet-derived factors to more effectively regulate the joint microenvironment. Collectively, these findings provide promising evidence that the compositional rationale behind increasing extraplatelet molecules in a PRP formulation confers measurable biological advantages in a multicellular OA model, supporting the rationale for further development and clinical investigation of compositionally optimized PRP preparations.

Several limitations of the present study must be acknowledged. The pooling strategy employed, combining 32 blood donors divided into four pools of eight donors each, ensured sufficient biological material for the full experimental workflow, but necessarily reduces statistical power and may mask inter-individual variability in PRP composition and cellular response. Future studies should incorporate larger cohorts with individual-level analysis to confirm the consistency of these findings across donors. In addition, extraplatelet molecules such as HGF or IGF-1 were not directly quantified in the specific plasma pools used for these in vitro experiments. Instead, the association between sPRP, BPCP, and extraplatelet enrichment relies on previous biochemical characterizations of the formulation. Therefore, while the enhanced biological activity observed for BPCP is consistent with an enrichment in extraplatelet bioactive molecules, further studies including direct biochemical profiling of each experimental batch and PRP formulation are warranted to establish definitive dose–response correlations. Furthermore, the biochemical characterization of the formulations would serve to determine the number of proteins secreted into the medium by the cells after inflammation. Gene expression analysis was restricted to hCh; extending RT-qPCR to hSv in subsequent studies will be important to determine whether the transcriptional effects of PRP differ between the two cell types and to obtain a more complete picture of the transwell co-culture response [57]. Additionally, the use of primary hCh and hSv derived from a single donor represents another limitation of the study. Although this approach was deliberately adopted to minimize donor-dependent variability and to establish a highly standardized model for this initial screening study, it does not account for the well-recognized biological heterogeneity that exists among individuals. Future studies analyzing cells from multiple donors will be necessary to assess the reproducibility of these findings. Another limitation is the use of IL-1β alone as an inflammatory inducer. Other molecules, such as TNF-α or LPS, generate an inflammatory response that activates different molecular pathways, which would be interesting to study in the future. In turn, the study focused on the inflammatory stage, which is part of the early phase of OA. For this reason, it would be interesting to examine the acute phase of the disease in future studies. From a model perspective, the transwell system captures paracrine signaling but does not replicate the full complexity of the OA joint, which involves additional cell types as well as mechanical loading and 3D matrix interactions. Validation in more sophisticated platforms, such as 3D co-culture scaffolds, organ-on-chip systems, or in vivo models, will be necessary to confirm the translational relevance of these findings.

Taken together, the present findings demonstrate that PRP treatment, and possibly BPCP formulation, exerts a consistent modulatory effect on inflammation, oxidative stress, and key catabolic markers in a multicellular OA-like in vitro model. The discrepancies observed between gene expression and protein secretion data are likely to reflect both the complex temporal dynamics of these regulatory processes and the limited statistical power inherent to the pooled experimental design. Rather than undermining the biological relevance of the findings, these discrepancies highlight the multifaceted nature of PRP’s mechanism of action and reinforce the need for future studies with larger cohort sizes, extended treatment windows, and complementary omics approaches to fully resolve the regulatory landscape of PRP in the OA joint multicellular environment.

## 4. Materials and Methods

### 4.1. Sample Collection and Preparation

#### 4.1.1. Donors

Thirty-two healthy volunteers aged between 21 and 65 years were selected for this study. Blood was collected in 9 mL tubes containing 3.8% (*w*/*v*) sodium citrate. Whole blood collected from thirty-two donors was then centrifuged to obtain plasma. Plasma samples were combined to generate four independent pooled preparations (n = 4, 8 donors per pool). sPRP and BPCP were prepared from the same donors. This research was carried out following the principles outlined in the Declaration of Helsinki and was approved by the Institutional Ethics Committee of OSI Araba (approval number 2024-023, dated 30 May 2024). Written informed consent was obtained from all participants prior to their involvement in the study.

#### 4.1.2. Standard Platelet-Rich Plasma Preparation

Briefly, the sPRP was obtained using a commercially available PRP kit (BTI Biotechnology Institute, Vitoria-Gasteiz, Spain) by centrifuging 9 mL of blood at 580× *g* for 8 min at room temperature (RT). The 2 mL plasma fraction present over the red blood cell fraction was collected, avoiding white blood cells from the ‘buffy coat’ layer. Obtained plasma fractions were randomly assigned into four pools (n = 4), each comprising samples from eight individuals. Platelet levels were measured in each pool. Then, 10% CaCl_2_ (20 μL/mL) [58], as reported in other studies, was added to the sPRP formulation for the release of platelet content and clot formation at 37 °C. The resulting sPRP releasate was sterilized by filtration through a Minisart^®^ NML Plus 0.2 μm filter (Sartorius, Goettingen, Germany). Finally, plasma was aliquoted and immediately stored at −80 °C for later analysis.

#### 4.1.3. Balanced Protein-Concentrate Plasma Preparation

As described by Mercader et al. [29], the BPCP was obtained by centrifuging 9 mL of whole blood at 1200× *g* for 8 min at RT, collecting the entire plasma column (PC). PCs were combined to generate four independent pooled preparations (n = 4), consisting of eight donors per group. Platelet levels were measured in each pool. The obtained pooled PCs contained levels of platelets and circulating molecules similar to blood basal levels, so this plasma fraction was employed as a reference control, providing the baseline platelet concentration against which the experimental samples were compared.

For the preparation of BPCP, PC was put in contact with 0.125 g/mL of hydroxyethyl acrylamide (HEAA) hydrogel, and after 5 min the PC water content was absorbed. The hydrogel powder was discarded by putting the plasma in contact with a 100 µm filtration unit on top of a 50 mL falcon tube. Centrifugation at 500× *g* for 2 min at RT was performed to collect the BPCP. Platelet levels were measured in each pool. Finally, 10% CaCl_2_ (20 µL/mL) [58], as reported in other studies, was added to the BPCP formulation, and it was kept at 37 °C for the release of platelet content. The resulting BPCP releasate was sterilized by filtration through a Minisart^®^ NML Plus 0.2 μm filter. Finally, BPCP releasate was aliquoted and stored immediately at −80 °C for later analysis.

### 4.2. In Vitro Model of OA

#### 4.2.1. Human Chondrocyte Cell Cultures

Commercially available hCh cells were derived from a 22-year-old male donor (P10970, Innoprot, Derio, Bizkaia, Spain), and cell culture was performed according to the manufacturer’s instructions. Briefly, cells were expanded in T-75 culture flasks in chondrocyte medium (CM, P60137; Innoprot, Derio, Bizkaia, Spain), supplemented with 2.5% (*v*/*v*) fetal bovine serum (FBS), Chondrocyte Growth Supplement (CGS), and penicillin/Streptomycin (P/S) at 1% (*v*/*v*) each. Cells were maintained at 37 °C in a humid atmosphere containing 5% CO_2_ up to passage 3.

#### 4.2.2. Human Synoviocyte Cell Cultures

Commercially available primary hSv were derived from healthy human synovium (P10972, Innoprot, Derio, Bizkaia, Spain) and cell culture was performed according to the manufacturer’s instructions. Briefly, cells were expanded in T-75 culture flasks in a synoviocyte medium (SM, P60127; Innoprot, Derio, Bizkaia, Spain), supplemented with 2% (*v*/*v*) FBS, Synociocyte Growth Supplement (SGS) and P/S at 1% (*v*/*v*) each. Cells were maintained at 37 °C in a humid atmosphere containing 5% CO_2_ up to passage 3.

#### 4.2.3. Transwell Co-Culture of hCh and hSv Cells

hCh and hSv cells were co-cultured in a transwell system [36]. hCh cells were seeded (±50,000 cells/well) in a 24-well plate (Costar, Corning, Corning, NY, USA) pre-treated with 0.1 mg/mL Poly-L-Lysine (P0879-25MG, Merck, Darmstadt, Germany) for 24 h to reach approximately 80% confluence. After reaching this status, hSv cells were seeded onto PET membrane inserts with a 0.4 μm pore size (9320402, CellQART, Northein, Germany) at a density of 12,500 cells/insert for 24 h. Then, the co-culture was established in CM supplemented with 10% PRP releasate (sPRP or BPCP). A total of 800 µL of medium was added to each well of the 24-well plate, and 200 µL to the upper transwell insert. IL-1β (10 ng/mL; 200-01B-2UG, Thermo Fisher Scientific, Waltham, MA, USA) was added together with the plasma treatment, as described in other studies on inflammation [59,60,61]. Cells cultured in serum-free medium (SF) stimulated together with IL-1β served as the experimental control, whereas cells cultured in SF without IL-1β were used as the negative control. The cells were incubated under treatment for 48 h at 37 °C in a humidified atmosphere with 5% CO_2_.

### 4.3. Gene Expression Analysis

Gene expression analysis was carried out for hCh cells. After 48 h of transwell co-culture, hCh cells were lysed using 1 mL of TRIzol (15596026, Thermo Fisher Scientific, Waltham, MA, USA). Total RNA extraction, quantification, and quantitative reverse transcription PCR (RT-qPCR) from cell cultures were performed by the Genetics, Epigenetics and Cellular Biology platform of the Bioaraba Health Research Institute (Vitoria-Gasteiz, Spain).

#### 4.3.1. Total RNA Extraction, Quantification and Quality Control

Total RNA was extracted from cells stored in TRIzol using the Direct-zol™ RNA Miniprep kit (Zymo Research, Tustin, CA, USA), following the manufacturer’s instructions. Briefly, cells were thawed and carefully homogenized, and RNA was purified using silica columns included in the kit, which allow the removal of genomic DNA and organic contaminants without the need for alcohol precipitation. In addition, during the purification process, samples were treated with DNase I to ensure that the isolated RNA was intact and free of DNA. The RNA was eluted in nuclease-free water and stored at −80 °C until use.

RNA concentration was determined using the Qubit™ RNA HS Assay Kit (Thermo Fisher Scientific, Waltham, MA, USA) using a Qubit™ 3.0 fluorometer. RNA purity and quality were further assessed by spectrophotometry with NanoDrop™ One C (Thermo Fisher Scientific, Waltham, MA, USA), considering the A260/280 and A260/230 ratios to estimate sample purity.

#### 4.3.2. Quantitative Reserve Real-Time PCR (RT-qPCR)

Gene expression was analyzed by two-step RT-qPCR. First, total RNA was reverse-transcribed using the High-Capacity cDNA Reverse Transcription Kit with RNase Inhibitor (Applied Biosystems, Thermo Fisher Scientific, Waltham, MA, USA), following the manufacturer’s recommendations. Up to 1 µg of total RNA was used as a template in 20 µL reactions under the following conditions: 25 °C for 10 min, 37 °C for 2 h, and 85 °C for 5 min for enzyme inactivation. The cDNA obtained was immediately used in quantitative amplification.

qPCR was performed with TaqMan™ Universal PCR Master Mix (Applied Biosystems) in a 7500 Real-Time PCR System (Applied Biosystems, Thermo Fisher Scientific, Waltham, MA, USA), using custom-designed plates (Custom TaqMan™ Array Plates, Thermo Fisher Scientific, Waltham, MA, USA) that included specific probes for the genes of interest (Supplementary Materials, Table S1) as well as negative controls (GAPDH, HPRT, and GUSB) to verify the absence of contamination. Each qPCR reaction had a final volume of 20 µL.

The amplification program consisted of an initial denaturation at 95 °C for 10 min, followed by 40 cycles of 95 °C for 15 s and 60 °C for 1 min. Ct values were obtained automatically using Applied Biosystems 7500 v2.0.3 software, and relative expression was calculated using the ΔΔCt method, using housekeeping genes for normalization.

### 4.4. Biochemical Analysis

#### 4.4.1. Platelet Level Measurement

Platelet levels were measured in PC, sPRP, and BPCP using a Mindray BC-20s hematology analyzer (Mindray, Shenzhen, China).

#### 4.4.2. Analysis of Secreted Proteins

Several cytokines and protein levels were detected in co-culture supernatant by an immunology Multiplex assay using different commercially available MILLIPLEX^®^ panels following the manufacturer’s instructions. IL-1β, TNF-α, IL-4, IL-6, IL-8, IL-10, MCP-1, MMP-1, MMP-7, and MMP-9 were analyzed using HPLX1-115SP-10M PLEXpedition Screening panel MIXMATCH (Merck, Darmstadt, Germany). MMP-3 and MMP-13 levels were measured by HMMP1MAG-55K-02 Human MMP Panel 1 Magnetic (Merck, Darmstadt, Germany). Plates were analyzed by a Luminex xMAP analyzer (Thermo Fisher Scientific, Waltham, MA, USA). Technical duplicates of all plasma samples were carried out. All protein levels were measured by fluorescence, and the corresponding concentrations were calculated by means of calibration curves (Four-Parameter Logistic Regression, 4PL).

### 4.5. ROS Level Measurement

Intracellular reactive oxygen species (ROS) were detected by the Cellular ROS Assay Kit (ab113851, Abcam, Burlingame, CA, USA) following the product information sheet after 48 h of cell culture. It includes the cell-permeant reagent 2′,7′-dichlorofluorescein diacetate (DCFDA), which is oxidized by intracellular ROS into a green fluorescent product.

Given the technical requirements of the DCFDA-based ROS assay, hCh and hSv cells were directly co-cultured (±1.5 × 10^4^ cells/well) in 96-well plates in complete CM (100 µL) for 24 h. Then, cells were exposed to 10 ng/mL IL-1β (200-01B-2UG, Thermo Fisher Scientific, Waltham, MA, USA) in fresh CM at 37 °C for 24 h. After incubation, cells were washed and exposed to 30 µM DCFDA at 37 °C for 45 min in the dark. Unexposed cells to DCFDA served as the negative control. After washing, cells were treated with Buffer 1× supplemented with 10% (*v*/*v*) sPRP or BPCP releasate and incubated at 37 °C for 4 h. Cells treated with the SF condition were used as a control. In addition, cells treated without the probe were used to determine the background of the treatment, which was subtracted from each measured value. Finally, stained cells were observed by a fluorescence Carl ZeissTM Axio Vert A1 inverted optical microscope (Zeiss, Oberkochen, Germany), and fluorescence intensity was measured using a TECAN Infinite 200 PRO plate reader (TECAN, Zurich, Switzerland) at 485/535 nm. TBHP in Buffer 1× (100 µM) was used as the positive control of the assay. Technical triplicates of all plasma samples were carried out.

### 4.6. Statistical Analysis

The distribution of the data was assessed by Shapiro–Wilk’s normality test. The different variables are presented as the mean ± SD for parametric data. The statistical significance of the differences between two groups was determined with Student’s *t*-test. The difference among more than two groups was validated with one-way or two-way ANOVA followed by Tukey/Dunnett’s multiple comparison test. Nevertheless, for non-parametric data, Welch ANOVA test was performed. Data were considered statistically significant when *p* < 0.05. GraphPad Prism^®^ version 9.5 (GraphPad Software, San Diego, CA, USA) was used for the statistical analyses.

## 5. Conclusions

Both PRP releasates modulated the inflammatory and oxidative microenvironment in a human chondrocyte–synoviocyte co-culture model at multiple biological levels, attenuating pro-inflammatory mediators, suppressing catabolic MMP secretion, and reducing intracellular ROS under IL-1β-induced conditions. The transwell co-culture system proved a valid platform for evaluating PRP bioactivity in an in vitro multicellular OA-relevant context. Notably, BPCP demonstrated a trend toward a more pronounced regulatory capacity at the protein level. These findings support compositional optimization as a key determinant of PRP efficacy beyond platelet concentration alone and warrant validation in more complex translational models.

## Figures and Tables

**Table 1:** Summary of characteristics for sPRP and BPCP pools.

	sPRP	BPCP
**1. PRP Preparation**		
Initial blood volume	9 mL per tube	9 mL per tube
Anticoagulant	Sodium citrate 3.8% (wt/V)	Sodium citrate 3.8% (wt/V)
System	Close	Open
Centrifugation	Yes	Yes
Number	1	1
Speed	580 *g*—8 min	1200 *g*—8 min
Water absorption	No	Yes
Method		HEAA hydrogel
Hydrogel concentration		0.125 g/mL
Contact time		5 min
Final PRP volume	2 mL per tube	2 mL per subject
**2. PRP Characteristics**		
PRP type	24-00-11	24-00-11
Platelets	452 × 10^3^/µL ± 63.38	468 × 10^3^/µL ± 60.65
Red blood cells	<0.01 × 10^6^/µL	<0.01 × 10^6^/µL
White blood cells	<0.05 × 10^6^/µL	<0.05 × 10^6^/µL
Neutrophils	---	---
Lymphocytes	---	---
Monocytes	---	---
Eosinophils	---	---
Basophils	---	---
Activation	CaCl_2_ (10% wt/vol)	CaCl_2_ (10% wt/vol)
**3. Application Characteristics**		
Dose	10%	10%
Direct/indirect	Direct	Direct
**4. Other remarkable PRP and study features**		
The product added to the cell cultures was the platelet releasate obtained following the activation of PRP with CaCl_2_ (10%). sPRP and BPCP doubled the basal blood levels of platelets.		

sPRP: Standard platelet-rich plasma; BPCP: Balanced protein-concentrate plasma; HEAA: Hydroxyethyl acrylamide.

## Data Availability

Data will be made available on request.

## Supplementary Materials

The following supporting information can be downloaded at: https://www.mdpi.com/article/10.3390/ijms27208950/s1.

## Abbreviations

The following abbreviations are used in this manuscript:

ANOVA	Analysis of variance
BPCP	Balanced protein-concentrate plasma
CG	Chondrocyte growth supplement
CM	Chondrocyte medium
DCFDA	2′,7′-Dichlorofluorescein diacetate
ECM	Extracellular matrix
ERDF	European Regional Development Fund
FBS	Fetal bovine serum
HEAA	Hydroxyethyl acrylamide
hCh	Human chondrocytes
HPRT	Hypoxanthine phosphoribosyltransferase
hSv	Human synoviocytes
OA	Osteoarthritis
P/S	Penicillin/streptomycin
PC	Plasma column
PET	Polyethylene terephthalate
PRP	Platelet-rich plasma
qPCR	Quantitative polymerase chain reaction
RNA	Ribonucleic acid
ROS	Reactive oxygen species
RT	Room temperature
RT-qPCR	Reverse transcription quantitative polymerase chain reaction
SD	Standard deviation
SF	Serum-free medium
SGS	Synoviocyte growth supplement
SM	Synoviocyte medium
sPRP	Standard platelet-rich plasma
TBHP	tert-butyl hydroperoxide
UCS	Universal coding system
xMAP	xMAP technology
