# Supplementary material for: Extraplatelet Molecule Enrichment Enhances the Ability of Platelet-Rich Plasma Releasate to Buffer IL-1β-Induced Inflammation and Oxidative Stress in a Primary Human Chondrocyte–Synoviocyte Co-Culture In Vitro System of Osteoarthritis
Source: Int J Mol Sci. 2026 Oct 9;27(20):8950. doi: 10.3390/ijms27208950 (PMC13704136; doi:10.3390/ijms27208950)
Supplement: Supplementary file 1 [file ijms-27-08950-s001.zip › ijms-4587259-Supplementary material_Materials&Methods_R1.pdf]

Supplementary material: materials and methods

1. Gene expression Analysis

1.1. Total RNA extraction, quantification and quality control

Total RNA was extracted from cells stored in TRIzol using the Direct-zol™ RNA Miniprep kit (Zymo Research, USA), following the manufacturer's instructions. Briefly, cells were thawed and carefully homogenized, and RNA was purified using silica columns included in the kit, which allow the removal of genomic DNA and organic contaminants without the need for alcohol precipitation. In addition, during the purification process, samples were treated with DNase I to ensure that the isolated RNA was intact and free of DNA. The RNA was eluted in nuclease-free water and stored at −80°C until use.

RNA concentration was determined using the Qubit™ RNA HS Assay Kit (Thermo Fisher Scientific, Massachusetts, USA), using a Qubit™ 3.0 fluorometer. RNA purity and quality were further assessed by spectrophotometry with NanoDrop™ One C (Thermo Fisher Scientific, Massachusetts, USA), considering the A260/280 and A260/230 ratios to estimate sample purity.

1.2. Quantitative Reserve Real-Time PCR (RT-qPCR)

Gene expression was analyzed by two-step RT-qPCR. First, total RNA was reverse transcribed using the High-Capacity cDNA Reverse Transcription Kit with RNase Inhibitor (Applied Biosystems, Thermo Fisher Scientific, Massachusetts, USA), following the manufacturer's recommendations. Up to 1 µg of total RNA was used as a template in 20 µL reactions under the following conditions: 25°C for 10 min, 37°C for 2 hours, and 85 °C for 5 min for enzyme inactivation. The cDNA obtained was immediately used in quantitative amplification.

qPCR was performed with TaqMan™ Universal PCR Master Mix (Applied Biosystems) in a 7500 Real-Time PCR System (Applied Biosystems, Thermo Fisher Scientific, Massachusetts, USA), using custom-designed plates (Custom TaqMan™ Array Plates, Thermo Fisher Scientific, Massachusetts, USA) that included specific probes for the genes of interest (Supplementary Information, Table 1) as well as negative controls (GAPDH, HPRT, GUSB) to verify the absence of contamination. Each qPCR reaction had a final volume of 20 μL.

The amplification program consisted of an initial denaturation at 95°C for 10 min, followed by 40 cycles of 95°C for 15 seconds and 60°C for 1 min. Ct values were obtained automatically using Applied Biosystems 7500 v2.0.3 software, and relative expression was calculated using the ΔΔCt method, using housekeeping genes for normalization.

1.3. ROS level measurement protocol

Given the technical requirements of the DCFDA-based ROS assay, hCh and hSv cells were directly co-cultured (± 1.5×10^4 cells/well) in 96-well plates in complete CM (100 μL) for 24 h, at a 4:1 hCh:hSv ratio. Then, cells were exposed to 10 ng/mL IL-1β (200-01B-2UG, Thermo Fisher Scientific, Massachusetts, EEUU) in fresh CM at 37˚C for 24 h. After incubation, cells were washed and exposed to 30 μM DCFDA at 37˚C for 45 min in the dark. Unexposed cells to DCFDA served as the negative control. After washing, cells were treated with Buffer 1X supplemented with 10% (v/v) sPRP or BPCP and incubated at 37°C for 4 h. Cells treated with SF condition were used as negative control. Finally, stained-cells were observed by a fluorescence Carl ZeissTM Axio Vert A1 inverted optic microscope (Zeiss, Oberkochen, Germany), and fluorescence intensity was measured using a TECAN Infinite 200 PRO plate reader (TECAN, Zurich, Switzerland)

at 485/535 nm. TBHP in Buffer 1x (100 μM) was used as the positive control of the assay. Technical triplicates of all plasma samples were carried out.
