# Supplementary material for: Extraplatelet Molecule Enrichment Enhances the Ability of Platelet-Rich Plasma Releasate to Buffer IL-1β-Induced Inflammation and Oxidative Stress in a Primary Human Chondrocyte–Synoviocyte Co-Culture In Vitro System of Osteoarthritis
Source: Int J Mol Sci. 2026 Oct 9;27(20):8950. doi: 10.3390/ijms27208950 (PMC13704136; doi:10.3390/ijms27208950)
Supplement: Supplementary file 1 [file ijms-27-08950-s001.zip › ijms-4587259-Supplementary material_Tables_R1.pdf]

Supplementary material

Table S1: Primer sequences used for RT-qPCR. All primers were acquired from Thermofisher Scientific, as well as custom-designed plates.

Gene	Thermofisher transcript	Thermofisher assay
**ACAN**	NM_001135.3/ NM_013227.3	Hs00153936_m1
**ADAMTS4**	NM_005099.5	Hs00192708_m1
**ADAMTS5**	NM_007038.4	Hs01095518_m1
**Bax**	NM_001291428.1	Hs00180269_m1
**Bcl-2**	NM_000633.2	Hs04986394_s1
**Casp3**	NM_004346.3	Hs00234387_m1
**Casp9**	NM_001229.4	Hs00962278_m1
**COL1A1**	NM_000088.3	Hs00164004_m1
**COL2A1**	NM_001844.4	Hs00264051_m1
**COX-2 (PTGS2)**	NM_000963.3	Hs00153133_m1
**IL-10**	NM_000572.2	Hs00961622_m1
**IL-13**	NM_002188.2	Hs00174379_m1
**IL1-B**	NM_000576.2	Hs01555410_m1
**IL-4**	NM_000589.3	Hs00174122_m1
**IL-6**	NM_000600.4	Hs00174131_m1
**IL-8 / CXCL8**	NM_000584.3	Hs00174103_m1
**iNOS**	NM_000625.4	Hs01075529_m1
**MCP-1**	NM_002982.3	Hs00234140_m1
**MMP-1**	NM_001145938.1	Hs00899658_m1
**MMP-13**	NM_002427.3	Hs00942584_m1
**MMP-3**	NM_002422.4	Hs00968305_m1
**MMP-7**	NM_002423.4	Hs01042796_m1
**MMP-9**	NM_004994.2	Hs00957562_m1
**NFKBIA (Ikb-α)**	NM_020529.2	Hs00355671_g1
**RELA (nfkb)**	NM_021975.3	Hs01042014_m1

**Sox9**	NM_000346.3	Hs00165814_m1
**TNF-α**	NM_000594.3	Hs00174128_m1
**GAPDH**	NM_001289746.1	Hs99999905_m1
**HPRT**	NM_000194.2	Hs99999909_m1
**GUSB**	NM_000181.3	Hs99999908_m1

Table S2: Protein expression analysis in cell culture media of hCh and hSv co-culture following treatment with sPRP and BPCP formulations under no inflammatory environment. Error bars = standard deviation (n = 4). Statistically significant differences were calculated using one-way ANOVA. Statistics marked in bold represent statistical significance between groups (A, B or C).

Protein	Protein levels (ng/mL)			p-value		
	SF (A)	sPRP (B)	BPCP (C)	A*B	A*C	B*C
**IL-1β**	1.182	2.13	3.02	0.0538	**0.0021**	**0.0311**
**TNF-α**	0.944	5.87	8.93	**0.0025**	**0.0001**	**0.0113**
**IL-6**	19.818	1210.65	4059.05	0.2334	**0.0012**	**0.0027**
**IL-8**	270.472	2447.71	3227.38	**0.0003**	**<0.0001**	**0.03**
**MCP-1**	336.784	1705.97	2138	**0.0003**	**<0.0001**	**0.0446**
**IL-4**	0.22	0.14	0.21	0.1661	0.9795	0.1218
**MMP-1**	518.646	904.0855	622.07	**0.0097**	0.1471	**0.0237**
**MMP-3**	21681.045	77.79.5	117542.1	**0.0013**	**0.0152**	0.1534
**MMP-7**	396.65	990.65	1531.884	**0.0106**	**0.0018**	**0.0073**
**MMP-9**	124.848	1224.96	2584.94	**0.0074**	**0.0002**	**0.0012**
**MMP-13**	-	-	-	-	-	-

Table S3: Protein expression analysis in the conditioned media of hCh/hSv co-cultures simultaneously treated with IL-1β (10 ng/mL) and sPRP or BPCP formulations. Error bars = standard deviation (n = 4). IL-1β stimulation and plasma treatment were applied simultaneously and maintained throughout the same incubation period (48 h). Statistically significant differences were calculated using one-way ANOVA. Statistics marked in bold represent statistical significance between groups (A, B or C).

Protein	Protein levels (ng/mL)			p-value		
	SF (A)	sPRP (B)	BPCP (C)	A*B	A*C	B*C
**IL-1β**	6352.82	5183.29	4781.47	**0.0079**	**0.0015**	0.225
**TNF-α**	20.56	16.53	17.45	0.0527	0.13	0.705
**IL-6**	32594.17	32207.61	27439.54	0.97	0.0591	**0.0355**
**IL-8**	2999.33	3769.72	3894.15	0.1553	0.0971	0.909
**MCP-1**	3205.31	3260.53	2422.65	0.983	0.188	0.0811
**IL-4**	1.4	2.13	1.45	0.7395	0.9608	0.8268
**MMP-1**	7682.2	4488.6	3252.79	**0.0028**	**0.0006**	**0.0461**
**MMP-3**	500839.15	486871.71	485822.25	0.4274	0.4343	0.9996
**MMP-7**	353.18	918.02	1549.883	**<0.0001**	**0.0019**	**0.0131**
**MMP-9**	135.71	1252.78	2755.58	**0.0093**	**0.0002**	**0.0012**
**MMP-13**	425.08	134.48	193.14	**0.0009**	**0.0056**	0.1834
